# Dosimetric Impact of the Positional Imaging Frequency for Hypofractionated Prostate Radiotherapy – A Voxel-by-Voxel Analysis

**DOI:** 10.3389/fonc.2020.564068

**Published:** 2020-09-29

**Authors:** Mona Splinter, Ilias Sachpazidis, Tilman Bostel, Tobias Fechter, Constantinos Zamboglou, Christian Thieke, Oliver Jäkel, Peter E. Huber, Jürgen Debus, Dimos Baltas, Nils H. Nicolay

**Affiliations:** ^1^Heidelberg Institute of Radiation Oncology (HIRO), National Center for Radiation Research in Oncology (NCRO), Heidelberg, Germany; ^2^Medical Physics in Radiation Oncology, German Cancer Research Center, Heidelberg, Germany; ^3^Department of Radiation Oncology, University of Freiburg – Medical Center, Freiburg im Breisgau, Germany; ^4^German Cancer Consortium (DKTK), Partner Site Freiburg, German Cancer Research Center, Heidelberg, Germany; ^5^Clinical Cooperation Unit Radiation Oncology, German Cancer Research Center, Heidelberg, Germany; ^6^Department of Radiation Oncology, University Medical Center Mainz, Mainz, Germany; ^7^Department of Radiation Oncology, University Hospital, LMU Munich, Munich, Germany; ^8^Department of Radiation Oncology, Heidelberg University Hospital, Heidelberg, Germany

**Keywords:** prostate cancer, image-guided radiotherapy, hypofractionation, dosimetry, organs-at-risk, tumor control probability

## Abstract

**Background:**

To investigate deviations between planned and applied treatment doses for hypofractionated prostate radiotherapy and to quantify dosimetric accuracy in dependence of the image guidance frequency.

**Methods:**

Daily diagnostic in-room CTs were carried out in 10 patients in treatment position as image guidance for hypofractionated prostate radiotherapy. Fraction doses were mapped to the planning CTs and recalculated, and applied doses were accumulated voxel-wise using deformable registration. Non-daily imaging schedules were simulated by deriving position correction vectors from individual scans and used to rigidly register the following scans until the next repositioning before dose recalculation and accumulation. Planned and applied doses were compared regarding dose-volume indices and TCP and NTCP values in dependence of the imaging and repositioning frequency.

**Results:**

Daily image-guided repositioning was associated with only negligible deviations of analyzed dose-volume parameters and conformity/homogeneity indices for the prostate, bladder and rectum. Average CTV T did not significantly deviate from the plan values, and rectum NTCPs were highly comparable, while bladder NTCPs were reduced. For non-daily image-guided repositioning, there were significant deviations in the high-dose range from the planned values. Similarly, CTV dose conformity and homogeneity were reduced. While TCPs and rectal NTCPs did not significantly deteriorate for non-daily repositioning, bladder NTCPs appeared falsely diminished in dependence of the imaging frequency.

**Conclusion:**

Using voxel-by-voxel dose accumulation, we showed for the first time that daily image-guided repositioning resulted in only negligible dosimetric deviations for hypofractionated prostate radiotherapy. Regarding dosimetric aberrations for non-daily imaging, daily imaging is required to adequately deliver treatment.

## Introduction

Prostate cancer is the most common malignancy in men with an estimated incidence ranging between 100 and 170 per 100 000 people per year (1). Radiotherapy is a key treatment modality for prostate cancer patients that results in survival rates comparable to prostatectomy while exhibiting lower rates of urinary incontinence or erectile dysfunction (2–4). Modern treatment techniques including image-guided and intensity-modulated radiotherapy (IMRT) have contributed to reducing late therapy-related toxicities to the bladder and rectum and allowed application of higher fractional and overall treatment doses (5, 6). A deeper understanding about the radiobiology of prostate cancers has established treatment concepts utilizing increasing single doses, and several large randomized trials have investigated the clinical relevance of hypofractionated radiotherapy for prostate cancer treatment (7–9). However, the increasing utilization of high-precision radiation techniques for the treatment of prostate cancer has rendered the application more susceptible to dosimetric deviations from the treatment plan due to inter- and intrafractional alterations in the pelvic anatomy, and the application of higher single doses in a reduced number of treatment fractions may increase the potential for therapeutic misses (10, 11). Regular image guidance using orthogonal X-ray or cone-beam CT (CBCT) imaging is set to accommodate for anatomic changes, and due to the weak soft-tissue contrast of pelvic positional imaging, additional means for positional control such as implanted fiducial markers may help to further improve reproducible treatment positioning for prostate cancer patients.

While the anatomic changes during the course of therapy have been widely studied, the consequences of these changes for the applied radiation doses remain incompletely understood (10–12). The majority of previous publications was based on weekly CT scans and rigid co-registration algorithms and demonstrated significant dosimetric variability for normofractionated IMRT and proton radiotherapy (13–15). So far, very few data are available comparing planned and applied doses for prostate radiotherapy based on daily imaging and deformable imaging registration, and no information is available for hypofractionated treatment concepts (16, 17). Additionally, while many guidelines recommend daily positional imaging for hypofractionated prostate radiotherapy, the majority of patients in the randomized trials did not receive image-guided treatment (18, 19).

Here, we analyzed deviations of the applied treatment doses from the treatment plan on a voxel-by-voxel basis using daily planning CT scans in treatment position performed immediately before each treatment fraction. Additionally, the dosimetric impact of different frequencies for positional imaging was quantified. These data will help to define optimal imaging frequencies and to devise adaptive re-planning strategies for hypofractionated prostate radiotherapy.

## Materials and Methods

### Patients

Ten consecutive patients were treated with definitive prostate radiotherapy at the German Cancer Research Center and were included in this analysis. All patients presented with low or intermediate-risk prostate cancer limited to stages T1c to T2b, PSA values ranging below 20 ng/ml and Gleason scores not exceeding 7 (20). The analysis was carried out in accordance with the Declaration of Helsinki (Seventh Revision, 2013) and was approved by the Independent Ethics Committee of the Medical Faculty of the University of Heidelberg, Germany (S-380/2017).

### Treatment Planning and Delivery

Patient immobilization was carried out using a ProStep^TM^ pelvic and lower extremity support (Elekta, Stockholm, Sweden). No patient received implanted fiducials prior to radiotherapy. The clinical target volume (CTV) covered the prostate gland for low-risk tumors and also the base of the seminal vesicles for intermediate-risk cancers. The planning target volume (PTV) comprised an additional 7 mm as a setup margin. Treatment plans were generated based on the hypofractionated arm of the CHHiP trial to a total dose of 60 Gy in 20 fractions of 3 Gy (7). Dose constraints to the organs-at risk (OARs) were defined in accordance with the Quantitative Analyses of Normal Tissue Effects in the Clinic (21–23). Treatment plans for a step-and-shoot IMRT using 9 co-planar fields were generated on the RayStation planning system (RaySearch Laboratories, Stockholm, Sweden). All patients were instructed to present to each treatment fraction with a comfortably filled bladder and an empty rectum.

### In-Room CT Imaging

For each treatment fraction, patient positioning on the treatment couch was carried out as described above, and the couch was then rotated into a CT scanner (Primatom; Siemens OCS, Malvern, United States) directly adjacent to the linear accelerator. All patients received daily diagnostic CT imaging in treatment position as a means of position verification. The in-room CT scanner had been approved for treatment planning scans, and all scans were taken to the same specifications used for the individual planning examinations.

### Analysis of Variations and Dose Accumulation

All target volumes and OARs were contoured by a board-certified radiation oncologist according to current guidelines both on the planning CT scan and the daily in-room CT scans (24, 25). Daily position verification imaging was rigidly registered to the planning CTs as routinely done for patient repositioning prior to dose re-calculation. Applied fractional doses were calculated based on the daily imaging, and resulting dose distributions were mapped onto the planning CTs. Daily doses were accumulated on a voxel-by-voxel basis using the RayStation’s deformable image registration module, and dose comparison was carried out against the treatment plan (26). The quality and accuracy of the deformable registration was assessed by a board-certified radiation oncologist based on the prostate contours available for each scan.

Different non-daily imaging schedules were simulated by deriving the position correction vectors from the respective CT scans that was then used to rigidly register the following scans until the next simulated positional imaging to the planning CTs. For thrice weekly imaging, vectors were derived from treatment days 1, 3, 5, 6, 8, 10, 11, 13, 15, 16, 18, and 20. For simulation of a twice weekly imaging schedule, repositioning was simulated based on the scans from days 1, 4, 6, 9, 11, 14, 16, and 19. For weekly imaging simulation, repositioning relied on the scans from days 1, 6, 11, and 16. Applied treatment doses were compared to the planned dose distribution using established dose-volume indices including mean dose (D_*mean*_), doses to x% of total volume (D_*x*_) and volume at x Gy doses (V_*x*_). Dose conformity and homogeneity was quantified and compared using the conformity index (CI), the conformal index (COIN) and the homogeneity index (HI) for the total prescription dose as described previously, and dose uniformity was assessed using gEUD (16, 27, 28). The tumor control probabilities (TCP) for the CTV and PTV, the normal tissue complication probability (NTCP) for the bladder and rectum and the complication-free tumor control probability (P +) were calculated and considered for the analysis.

The applied dose distributions were compared within the region receiving >10% of the maximum dose using a 3D gamma analysis to the clinical tolerance level of 3%/3mm (29).

### Statistical Analysis

Differences in the dose volume indices between applied and prescribed doses were compared by the Wilcoxon signed-rank test with corresponding two-sided confidence intervals using an in-house software tool developed in Python^1^. Differences were considered statistically significant for *p* < 0.05.

## Results

### Impact of Interfractional Variations on Dose Distribution

**FIGURE 1:**
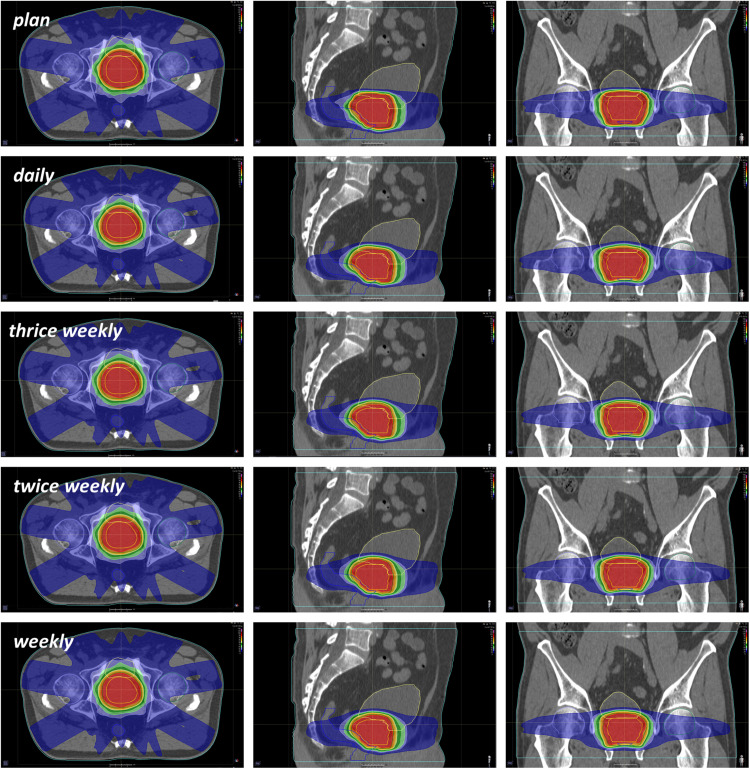
Representative CT images demonstrating the distribution of planned and applied doses in relation to the positional imaging frequency.

**FIGURE 2:**
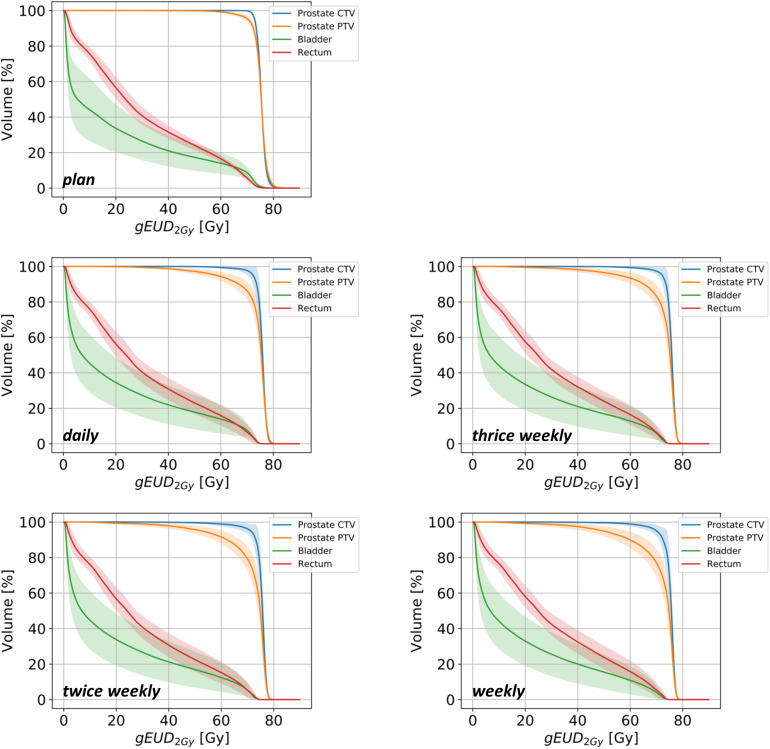
Summed dose-volume histograms for the prostate CTV (blue lines), PTV (orange lines), bladder (green lines) and rectum (red lines) for treatment plans and dose accumulations in dependency of the image-guided repositioning frequency. Lighter-colored bands represent the 95% confidence intervals for each curve.

**FIGURE 3:**
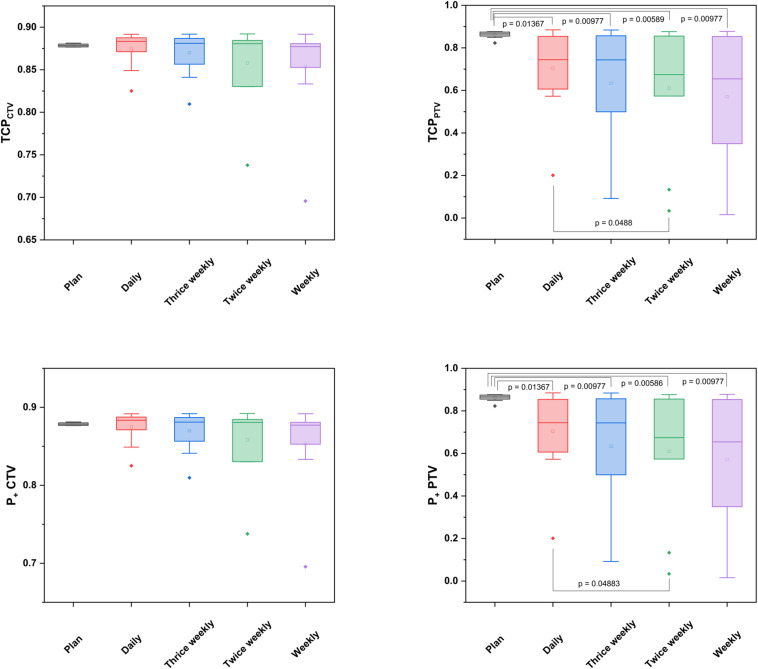
Box-plot diagrams for TCP and P + values of the CTVs and PTVs derived from the planned and accumulated doses in relation to the positional imaging frequency.

Deviations of the applied doses were compared with the treatment plan regarding the CTV, PTV, bladder and rectum (Figure 1). Due to its well-known changes in volume during radiotherapy, the bladder exhibited the highest variability between the applied and prescribed doses, and the dosimetric deviations were most pronounced in the higher-dose range for both the bladder and the rectum (Figure 2 and Supplementary Figure 1). The differences in the dose-volumetric indices between the accumulation of applied fractional doses and the treatment plan are summarized in Table 1. Upon daily rigid repositioning, the doses to the CTV did only demonstrate minor deviations ranging below 1% for all tested dose volume indices. The accumulated D_50_ and D_*mean*_ for the CTV deviated by only 0.3% from the planned doses (*p* = 0.160 for D_50_; *p* = 0.557 for D_*mean*_). No significant deviations were observed for the dose conformity indices of the CTV (*p* = 0.160 for CI, *p* = 0.084 for COIN), and a small decrease was only noted for dose homogeneity (*p* = 0.002 for HI, *p* = 0.846 for gEUD). As a result, the TCP and P + values for the CTV were comparable for the planned and applied treatment doses (87.8 vs. 88.1%; *p* = 0.695 for TCP; 81.5 vs. 81.5%; *p* = 0.625 for P +) (Figure 3). For the PTV, the differences were most pronounced in the low dose region with the applied D_98_ deviating by 20.5% (*p* = 0.002) compared to the planned D_98_. In the high-dose region, only the applied V_55_ deviated from the plan by 8.9% (*p* = 0.002), while the D_5_ and D_2_ values were comparable (*p* = 0.131 for D_5_; *p* = 0.064 for D_2_).

**TABLE 1:** Average and standard deviation of differences between applied and planned dose-volume indices for daily and different non-daily CT-based repositioning concepts. Negative values represent decreases in accumulated doses.

		Daily	*P* value	Thrice weekly	*P* value	Twice weekly	*P* value	Weekly	*P* value
**CTV**	D98 (Gy)	–0.008	0.770	–0.011	0.557	0.009	0.275	0.039	0.084
	D50 (Gy)	–0.003	0.160	–0.003	0.232	–0.003	0.275	–0.002	0.322
	Dmean (Gy)	–0.003	0.557	–0.003	0.846	–0.002	0.922	–0.001	0.492
	D5 (Gy)	0.003	0.492	0.004	0.322	0.007	0.193	0.008	0.105
	D2 (Gy)	0.005	0.322	0.008	0.131	0.010	0.131	0.011	0.084
	D1cc (Gy)	0.004	0.375	0.009	0.557	**0.039**	0.037*	**0.066**	0.014*
	V55 (%)	0.000	0.281	0.001	0.142	**0.002**	0.036*	**0.011**	0.014*
	gEUD (Gy)	–0.007	0.846	–0.004	1.000	0.000	0.181	0.003	0.275
	CI	–0.271	0.160	–0.259	0.160	–0.236	0.275	–0.216	0.275
	COIN	–0.358	0.084	−**0.600**	0.037*	−**0.667**	0.027*	−**0.581**	0.037*
	HI	**0.016**	0.002*	**0.019**	0.002*	**0.022**	0.002*	**0.023**	0.002*
**PTV**	D98 (Gy)	**0.205**	0.002*	**0.198**	0.002*	**0.253**	0.002*	**0.247**	0.002*
	D50 (Gy)	0.000	0.770	0.002	0.492	0.004	0.084	**0.006**	0.037*
	Dmean (Gy)	**0.019**	0.010*	**0.022**	0.010*	**0.034**	0.004*	**0.041**	0.004*
	D5 (Gy)	0.005	0.131	**0.009**	0.027*	**0.012**	0.014*	**0.013**	0.010*
	D2 (Gy)	0.008	0.064	**0.012**	0.006*	**0.015**	0.004*	**0.018**	0.002*
	D1cc (Gy)	**0.325**	0.010*	**0.312**	0.020*	**0.345**	0.014*	**0.323**	0.010*
	V55 (%)	**0.089**	0.002*	**0.105**	0.002*	**0.144**	0.002*	**0.158**	0.002*
	gEUD (Gy)	**0.268**	0.020*	**0.264**	0.014*	**0.308**	0.014*	**0.307**	0.010*
	CI	–0.003	0.846	0.084	0.275	0.122	0.084	0.172	0.064
	COIN	–0.003	0.695	0.082	0.232	0.162	0.064	0.201	0.064
	HI	**0.020**	0.002*	**0.029**	0.002*	**0.033**	0.002*	**0.033**	0.002*

		**Daily**	***P* value**	**Thrice weekly**	***P* value**	**Twice weekly**	***P* value**	**Weekly**	***P* value**

**Bladder**	D50 (Gy)	–0.398	0.131	–0.359	0.160	–0.489	0.084	–0.442	0.084
	Dmean (Gy)	–0.082	0.432	–0.006	0.695	–0.061	0.625	–0.044	0.770
	D25 (Gy)	–0.072	0.375	0.022	0.695	–0.056	0.375	–0.028	0.492
	D5 (Gy)	0.005	0.105	**0.022**	0.006*	**0.029**	0.006*	**0.044**	0.004*
	D1cc (Gy)	0.002	0.193	0.004	0.275	0.002	0.275	–0.002	0.275
	V55 (%)	–0.103	0.846	0.158	0.131	0.090	0.322	0.222	0.084
	V45 (%)	–0.120	0.695	0.004	0.625	–0.021	1.000	–0.064	0.695
	gEUD (Gy)	–0.020	0.770	**0.024**	0.049*	0.010	0.193	**0.040**	0.27*
**Rectum**	D50 (Gy)	0.050	0.625	0.022	0.275	0.041	0.375	0.024	0.131
	Dmean (Gy)	0.022	0.846	0.022	0.770	0.034	0.770	0.030	0.557
	D60 (Gy)	0.058	0.922	0.030	0.625	0.013	0.557	0.012	0.193
	D30 (Gy)	0.097	0.770	0.015	1.000	0.066	0.846	0.023	1.000
	D15 (Gy)	0.013	0.492	0.007	0.695	0.024	0.375	0.012	0.432
	D5 (Gy)	0.006	0.322	0.006	0.375	0.015	0.084	0.010	0.232
	D1cc (Gy)	–0.022	0.695	–0.062	0.375	0.009	0.557	–0.017	0.275
	V55 (%)	0.040	0.846	0.032	0.922	0.101	0.770	0.016	0.922
	V45 (%)	0.145	0.557	0.063	0.770	0.119	0.625	0.101	0.922
	gEUD (Gy)	0.015	0.492	–0.001	0.625	0.025	0.432	0.016	0.432

CTV, clinical target volume; gEUD, generalized equivalent uniform dose; CI, conformity index; COIN, conformal index; HI, homogeneity index. *p < 0.05. The asterisk and the bold print mark the significant values in table.

**FIGURE 4:**
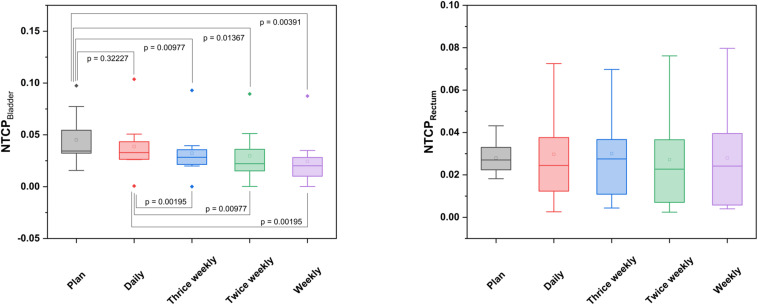
Box-plot diagrams for NTCP values of the bladder and rectum derived from the planned and accumulated doses in relation to the positional imaging frequency.

Considering the known alterations in bladder volumes during radiotherapy, the applied D_*mean*_ and D_50_ varied by 8.2 and 39.8% from the planned doses, respectively (*p* = 0.432 for D_*mean*_; *p* = 0.131 for D_50_); however, no statistically significant difference could be obtained for any tested dose-volume parameter. For the rectum, the accumulated average D_*mean*_ and D_50_ values deviated by only 2.2 and 5.0%, respectively (*p* = 0.846 for D_*mean*_; *p* = 0.625 for D_50_), and all tested dose-volume indices did only show non-significant variations from the treatment plan (Table 1). Similarly, the NTCP values derived from the accumulated or planned doses were comparable and did not significantly deviate for either the bladder (*p* = 0.322) or the rectum (*p* = 0.770) (Figure 4).

### Impact of Positional Imaging Frequency on Dose Distribution

To quantify the dosimetric consequences of various non-daily position verification CT schedules in comparison to daily positional imaging, CT-based correction vectors were derived on days 1, 3, 5, 6, 8, 10, 11, 13, 15, 16, 18, and 20 for the thrice weekly schedule, on days 1, 4, 6, 9, 11, 14, 16, and 19 for the twice weekly schedule and on days 1, 6, 11, and 16 for the weekly schedule, and were used to register all consecutive scans up to the next positional imaging onto the planning scan. Resulting applied doses were accumulated and compared to the dose accumulation resulting from daily CT-based repositioning.

For the CTV, no significant dosimetric deviations of the applied from the planned doses were observed, when positional imaging was simulated thrice weekly. For the twice weekly and weekly schedules, the V55 and D1cc deviated significantly from the treatment plan, while TCP and P + values were comparable (Table 1 and Figure 3). In contrast, for all non-daily imaging schedules, applied doses to the PTV deviated considerably from the planned doses: Alterations were most pronounced in the high-dose region with significant deviations of the D_5_ (*p* = 0.027 for thrice weekly, *p* = 0.014 for twice weekly, *p* = 0.010 for weekly imaging), the D_2_ (*p* = 0.006 for thrice weekly, *p* = 0.004 for twice weekly, *p* = 0.002 for weekly imaging), the D_1__*cc*_ (*p* = 0.020 for thrice weekly, *p* = 0.014 for twice weekly, *p* = 0.010 for weekly imaging) and the V_55_ (*p* = 0.002 for all non-daily imaging schedules).

For the bladder, small but significant differences between the applied and prescribed doses were only noted for the D_5_ in all non-daily imaging schedules (*p* = 0.006 for thrice weekly and twice weekly, *p* = 0.004 for weekly imaging), and the dose uniformity was reduced for thrice weekly (*p* = 0.049) and weekly CT-based positional correction (*p* = 0.027). All other dose volume parameters for the accumulated doses did not significantly deviate from the plan, likely due to a large inter-individual variability regarding bladder volumes (Table 1). The NTCP for the bladder was found significantly lower for all non-daily repositioning concepts in comparison to daily positional correction (*p* = 0.010 for thrice weekly, *p* = 0.014 for twice weekly, *p* = 0.004 for weekly imaging) (Figure 4). For the rectum, deviations of the accumulated fractional doses from the planned doses did not reach statistical significance for any tested dose volume parameter, and the NTCP values did not significantly deviate for any non-daily imaging algorithm.

**FIGURE 5:**
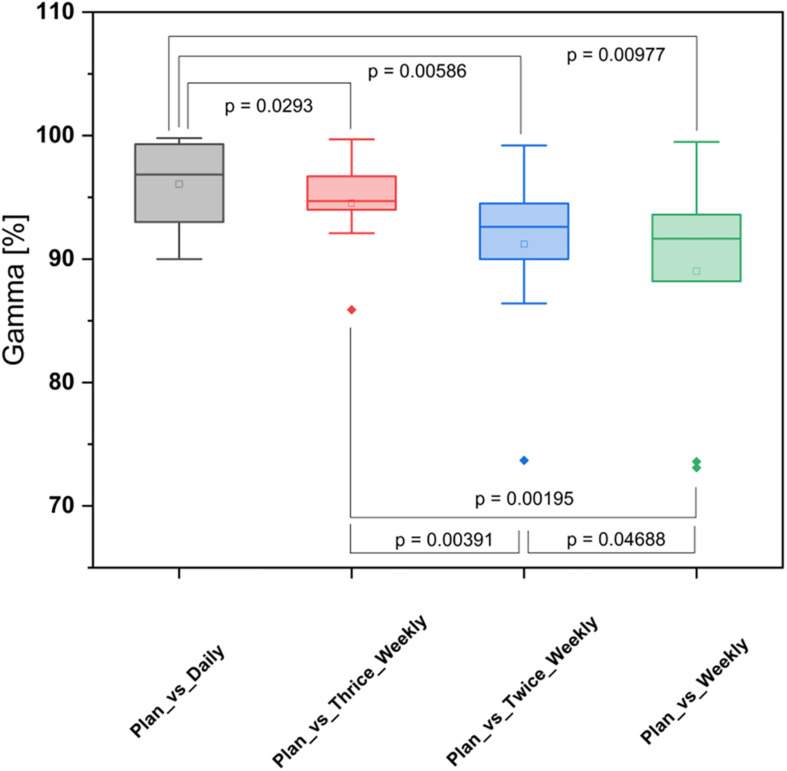
Box-plot diagram for the gamma analyses comparing different positional imaging frequencies.

The gamma passing rates to a clinical tolerance level of 3%/3mm were reduced by an average of 1.5% for thrice weekly imaging (*p* = 0.002), 4.9% for twice weekly imaging (*p* = 0.006) and 7.1% for weekly imaging (*p* = 0.010) (Figure 5).

## Discussion

Dosimetric deviations in prostate radiotherapy resulting from intra- and interfractional anatomic variability have been incompletely understood, and an in-depth comprehension about the dosimetric impact of position verification imaging becomes more important as individual fraction dose are increasing for hypofractionated or even stereotactic treatment approaches (18, 19, 30). Previous analyses have generally been limited by the insufficient quality or frequency of position verification imaging and the lacking availability of elastic image registration tools.

This dataset provides for the first time a comprehensive and voxel-based analysis of the dose deviations resulting from different imaging and repositioning frequencies for hypofractionated prostate radiotherapy. As daily planning quality CT scans and a state-of-the-art elastic image registration tool were used in this analysis, applied doses to the target volumes and organs-at risk could be precisely accumulated for each treatment fraction and each patient. Our data revealed that daily CT-based position correction did not lead to significant dosimetric deviations of the applied from the prescribed treatment doses irrespective of the daily pelvic anatomy. However, repositioning based on non-daily positional imaging resulted in significant dose differences especially in the high-dose range and diminished dose conformity and homogeneity to the target volume in case of hypofractionation.

Previous analyses have employed varying strategies to accumulate fractional doses for prostate cancer radiotherapy on the basis of widely available but low-quality CBCT imaging, including enhanced or iterative CBCT approaches, portal dose measurements or rigid registration strategies, albeit with limitations regarding dose mapping (12, 15, 31, 32). Dosimetric analyses based on daily diagnostic CT imaging have only been reported for proton radiotherapy, as in-room CT scanners are more widely available at the respective facilities, given the necessity for high-resolution imaging to compensate for higher proton range uncertainties due to anatomic alterations (33, 34).

The available evidence concerning the ideal frequency for positional imaging remains somewhat inconclusive. A previous publication reported improvements in target volume coverage and a reduction in high doses to the rectum for daily CBCT imaging and repositioning based on registration (35). It has been suggested that the imaging frequency should direct the choice of PTV margins, although the relevance of the observed dosimetric improvements for the treated patients remains unclear (36, 37). So far, several randomized trials have addressed the clinical relevance of daily CBCT regarding treatment-related toxicities and patient outcomes: A French trial enrolled 470 patients and reported improved progression-free survival and reduced late rectal toxicity for the cohort receiving daily CBCT. However, a Scandinavian trial failed to demonstrate any benefit of daily CBCT and PTV margin reduction regarding treatment-related toxicities or quality-of-life parameters in comparison to weekly portal imaging (38). For hypofractionated radiotherapy, it is conceivable that due to the increases in single doses, anatomy-related dosimetric deviations for individual treatment fractions may have a higher impact and may therefore warrant more frequent imaging as compared to normofractionation concepts. A substudy within the CHHiP trial analyzed the relevance of image guidance and PTV margin reduction for hypofractionated prostate radiotherapy and reported a borderline improvement of grade ≥2 late rectal toxicities only for image guidance and margin reduction, while standard-margin image-guided and non-image-guided therapies produced highly comparable results; bladder toxicities or patient outcomes appeared comparable in all three subgroups (18). The vast majority of patients in the CHHiP trial received 2D image guidance based on implanted fiducial markers, and the utilization of these markers may impact deviations of the applied doses from the treatment plan. However, fiducial markers seem to only result in insignificant dosimetric benefits in comparison to markerless registration strategies if daily CT imaging is available (12). The advent of hybrid MR-linear accelerators may further reduce anatomic and dosimetric deviations during the course of hypofractionated prostate radiotherapy, e.g., by utilizing the superior soft tissue contrast and the possibility for real-time prostate tracking: MR-guided imaging concepts would thereby allow to compensate for both interfractional and intrafractional motion (39, 40). In contrast to focusing on the dosimetric consequences of underlying anatomic pelvic alterations, novel position correction strategies have been proposed that guide repositioning based on the accumulated doses during treatment (41). The availability of voxel-wise information regarding accumulated doses in our dataset may help to further devise dose-directed repositioning strategies to compensate for already accumulated deviations during the course of radiotherapy.

Although our analysis is based on daily diagnostic-quality CT imaging and state-of-the-art elastic image registration and is therefore able to provide comprehensive voxel-wise dosimetric data, it has limitations: Beyond interfractional variability and setup errors, applied doses may also be influenced by intrafractional motion of the prostate that could not be quantified in our dataset. As out chosen methodology did not allow for intrafractional imaging, the impact of intrafractional variability for hypofractionated prostate radiotherapy needs to be addressed separately. All patients analyzed here received pre-treatment coaching about rectal and bladder filling as well as feedback in case of notable deviations of their pelvic anatomy from that of the planning CT. This coaching and a resulting consistency in rectal and bladder filling may have contributed to the relatively moderate dosimetric deviations observed in the simulated non-daily imaging schedules, and patients without coaching may in fact demonstrate significantly higher dosimetric deviations for non-daily imaging-guided repositioning. Additionally, generous PTV margins of 7 mm may at least have partly compensated for the deficiencies of all non-daily imaging schedules and hence to the reasonable TCP values observed. While the seminal CHHiP hypofractionation trial used PTV margins up to 10 mm, several newer clinical trials with daily positional imaging have employed smaller PTV margins ranging at around 5 mm (42, 43); the voxel-wise dosimetric data obtained from our analysis may help to devise concepts for PTV margin reduction that may in turn reduce treatment-related toxicities for hypofractionated prostate radiotherapy.

The transferability of our data to high-risk or locally advanced prostate cancers as well as other concepts of hypofractionation warrant further analyses, as differing treatment margins or treatment times may also influence dose-volume parameters.

Nevertheless, we provide for the first time a comprehensive and voxel-by-voxel analysis of the dosimetric effects of interfractional variations for hypofractionated prostate radiotherapy and the resulting consequences for the frequency of position verification imaging. These data will also help to devise strategies for adaptive planning of hypofractionated radiation treatments.

## Conclusion

Using voxel-by-voxel dose accumulation, we showed for the first time that daily image-guided repositioning resulted in only negligible dosimetric deviations for hypofractionated prostate radiotherapy. Regarding the observed dosimetric aberrations for the simulated non-daily imaging algorithms, daily imaging seems to be required to adequately deliver treatment. These data will help to develop adaptive re-planning strategies for hypofractionated prostate radiotherapy.

## Data Availability Statement

The datasets presented in this article are not readily available because German federal laws protect patient data and prohibit these data from being transferred or shared. Requests to access the datasets should be directed to nils.nicolay@uniklinik-freiburg.de.

## Ethics Statement

The studies involving human participants were reviewed and approved by Independent Ethics Committee of the University of Heidelberg. The patients/participants provided their written informed consent to participate in this study.

## Author Contributions

TB, PH, JD, and NN planned and carried out treatment. MS, IS, TB, TF, OJ, DB, and NN analyzed the data. NN wrote the manuscript. IS, CZ, CT, PH, and DB helped with writing the manuscript. JD helped with data discussion. All authors contributed to the article and approved the submitted version.

## Conflict of Interest

The authors declare that the research was conducted in the absence of any commercial or financial relationships that could be construed as a potential conflict of interest.

## Footnotes

1 https://www.python.org

## Supplementary Material

Supplementary Figure 1 Summed dose-volume histograms of the gEUD_2Gy_ for the prostate CTV (blue lines), PTV (orange lines), bladder (green lines) and rectum (red lines) for treatment plans and dose accumulations in dependency of the image-guided repositioning frequency. Lighter-colored bands represent the 95% confidence intervals for each curve.

## References

[1] Torre LA Siegel RL Ward EM Jemal A. Global cancer incidence and mortality rates and trends–an update. *Cancer Epidemiol Biomarkers Prev.* (2016) 25:16–27. 10.1158/1055-9965.EPI-15-0578 26667886

[2] Hamdy FC Donovan JL Lane JA Mason M Metcalfe C Holding P 10-Year outcomes after monitoring, surgery, or radiotherapy for localized prostate cancer. *N Engl J Med.* (2016) 375:1415–24. 10.1056/NEJMoa1606220 27626136

[3] Kupelian PA Potters L Khuntia D Ciezki JP Reddy CA Reuther AM Radical prostatectomy, external beam radiotherapy or =72 Gy, permanent seed implantation, or combined seeds/external beam radiotherapy for stage T1-T2 prostate cancer. *Int J Radiat Oncol Biol Phys.* (2004) 58:25–33. 10.1016/S0360-3016(03)00784-3 14697417

[4] Resnick MJ Koyama T Fan KH Albertsen PC Goodman M Hamilton AS Long-term functional outcomes after treatment for localized prostate cancer. *N Engl J Med.* (2013) 368:436–45. 10.1056/NEJMoa1209978 23363497 PMC3742365

[5] Viani GA Viana BS Martin JE Rossi BT Zuliani G Stefano EJ. Intensity-modulated radiotherapy reduces toxicity with similar biochemical control compared with 3-dimensional conformal radiotherapy for prostate cancer: a randomized clinical trial. *Cancer.* (2016) 122:2004–11. 10.1002/cncr.29983 27028170

[6] Bruner DW Hunt D Michalski JM Bosch WR Galvin JM Amin M Preliminary patient-reported outcomes analysis of 3-dimensional radiation therapy versus intensity-modulated radiation therapy on the high-dose arm of the radiation therapy oncology group (RTOG) 0126 prostate cancer trial. *Cancer.* (2015) 121:2422–30. 10.1002/cncr.29362 25847819 PMC4490066

[7] Dearnaley D Syndikus I Mossop H Khoo V Birtle A Bloomfield D Conventional versus hypofractionated high-dose intensity-modulated radiotherapy for prostate cancer: 5-year outcomes of the randomised, non-inferiority, phase 3 CHHiP trial. *Lancet Oncol.* (2016) 17:1047–60. 10.1016/S1470-2045(16)30102-4 27339115 PMC4961874

[8] de Vries KC Wortel RC Hoop EO Heemsbergen WD Pos FJ Incrocci PL. Hyprofractionated versus conventionally fractionated radiotherapy for patients with intermediate- or high-risk, localized, prostate cancer: 7-year outcomes from the randomized, multi-centre, open-label, phase 3 HYPRO trial. *Int J Radiat Oncol Biol Phys.* (2019) 106:108–15. 10.1016/j.ijrobp.2019.09.007 31593756

[9] Miralbell R Roberts SA Zubizarreta E Hendry JH. Dose-fractionation sensitivity of prostate cancer deduced from radiotherapy outcomes of 5,969 patients in seven international institutional datasets: alpha/beta = 1.4 (0.9-2.2) Gy. *Int J Radiat Oncol Biol Phys.* (2012) 82:e17–24. 10.1016/j.ijrobp.2010.10.075 21324610

[10] Verma V Chen S Zhou S Enke CA Wahl AO. Prostate bed target interfractional motion using RTOG consensus definitions and daily CT on rails : does target motion differ between superior and inferior portions of the clinical target volume? *Strahlenther Onkol.* (2017) 193:38–45. 10.1007/s00066-016-1077-6 27909738

[11] Bylund KC Bayouth JE Smith MC Hass AC Bhatia SK Buatti JM. Analysis of interfraction prostate motion using megavoltage cone beam computed tomography. *Int J Radiat Oncol Biol Phys.* (2008) 72:949–56. 10.1016/j.ijrobp.2008.07.002 19014783

[12] Wust P Joswig M Graf R Bohmer D Beck M Barelkowski T Dosimetric implications of inter- and intrafractional prostate positioning errors during tomotherapy : comparison of gold marker-based registrations with native MVCT. *Strahlenther Onkol.* (2017) 193:700–6. 10.1007/s00066-017-1141-x 28466155

[13] Zhang X Dong L Lee AK Cox JD Kuban DA Zhu RX Effect of anatomic motion on proton therapy dose distributions in prostate cancer treatment. *Int J Radiat Oncol Biol Phys.* (2007) 67:620–9. 10.1016/j.ijrobp.2006.10.008 17236979 PMC1945214

[14] Soukup M Sohn M Yan D Liang J Alber M. Study of robustness of IMPT and IMRT for prostate cancer against organ movement. *Int J Radiat Oncol Biol Phys.* (2009) 75:941–9. 10.1016/j.ijrobp.2009.04.032 19801105

[15] Moteabbed M Trofimov A Sharp GC Wang Y Zietman AL Efstathiou JA A prospective comparison of the effects of interfractional variations on proton therapy and intensity modulated radiation therapy for prostate cancer. *Int J Radiat Oncol Biol Phys.* (2016) 95:444–53. 10.1016/j.ijrobp.2015.12.366 26907917 PMC4834287

[16] Bostel T Sachpazidis I Splinter M Bougatf N Fechter T Zamboglou C Dosimetric impact of interfractional variations in prostate cancer radiotherapy-implications for imaging frequency and treatment adaptation. *Front Oncol.* (2019) 9:940. 10.3389/fonc.2019.00940 31612106 PMC6776888

[17] Splinter M Bostel T Sachpazidis I Fechter T Zamboglou C Jakel O Dosimetric impact of interfractional variations for post-prostatectomy radiotherapy to the prostatic fossa-relevance for the frequency of position verification imaging and treatment adaptation. *Front Oncol.* (2019) 9:1191. 10.3389/fonc.2019.01191 31788450 PMC6856079

[18] Murray J Griffin C Gulliford S Syndikus I Staffurth J Panades M A randomised assessment of image guided radiotherapy within a phase 3 trial of conventional or hypofractionated high dose intensity modulated radiotherapy for prostate cancer. *Radiother Oncol.* (2019) 142:62–71. 10.1016/j.radonc.2019.10.017 31767473 PMC7005673

[19] Wortel RC Heemsbergen WD Smeenk RJ Witte MG Krol SDG Pos FJ Local protocol variations for image guided radiation therapy in the multicenter dutch hypofractionation (HYPRO) trial: impact of rectal balloon and MRI delineation on anorectal dose and gastrointestinal toxicity levels. *Int J Radiat Oncol Biol Phys.* (2017) 99:1243–52. 10.1016/j.ijrobp.2017.07.044 28943074

[20] D’Amico AV Whittington R Malkowicz SB Schultz D Blank K Broderick GA Biochemical outcome after radical prostatectomy, external beam radiation therapy, or interstitial radiation therapy for clinically localized prostate cancer. *JAMA.* (1998) 280:969–74. 10.1001/jama.280.11.969 9749478

[21] Viswanathan AN Yorke ED Marks LB Eifel PJ Shipley WU. Radiation dose-volume effects of the urinary bladder. *Int J Radiat Oncol Biol Phys.* (2010) 76(3 Suppl):S116–22. 10.1016/j.ijrobp.2009.02.090 20171505 PMC3587780

[22] Michalski JM Gay H Jackson A Tucker SL Deasy JO. Radiation dose-volume effects in radiation-induced rectal injury. *Int J Radiat Oncol Biol Phys.* (2010) 76(3 Suppl):S123–9. 10.1016/j.ijrobp.2009.03.078 20171506 PMC3319467

[23] Marks LB Yorke ED Jackson A Ten Haken RK Constine LS Eisbruch A Use of normal tissue complication probability models in the clinic. *Int J Radiat Oncol Biol Phys.* (2010) 76(3 Suppl):S10–9. 10.1016/j.ijrobp.2009.07.1754 20171502 PMC4041542

[24] Salembier C Villeirs G De Bari B Hoskin P Pieters BR Van Vulpen M ESTRO ACROP consensus guideline on CT- and MRI-based target volume delineation for primary radiation therapy of localized prostate cancer. *Radiother Oncol.* (2018) 127:49–61. 10.1016/j.radonc.2018.01.014 29496279

[25] Boehmer D Maingon P Poortmans P Baron MH Miralbell R Remouchamps V Guidelines for primary radiotherapy of patients with prostate cancer. *Radiother Oncol.* (2006) 79:259–69. 10.1016/j.radonc.2006.05.012 16797094

[26] Motegi K Tachibana H Motegi A Hotta K Baba H Akimoto T. Usefulness of hybrid deformable image registration algorithms in prostate radiation therapy. *J Appl Clin Med Phys.* (2019) 20:229–36. 10.1002/acm2.12515 30592137 PMC6333149

[27] Feuvret L Noel G Mazeron JJ Bey P. Conformity index: a review. *Int J Radiat Oncol Biol Phys.* (2006) 64:333–42. 10.1016/j.ijrobp.2005.09.028 16414369

[28] Baltas D Kolotas C Geramani K Mould RF Ioannidis G Kekchidi M A conformal index (COIN) to evaluate implant quality and dose specification in brachytherapy. *Int J Radiat Oncol Biol Phys.* (1998) 40:515–24. 10.1016/S0360-3016(97)00732-3 9457842

[29] Clasie BM Sharp GC Seco J Flanz JB Kooy HM. Numerical solutions of the gamma-index in two and three dimensions. *Phys Med Biol.* (2012) 57:6981–97. 10.1088/0031-9155/57/21/6981 23044713 PMC3522748

[30] Bostel T Pfaffenberger A Delorme S Dreher C Echner G Haering P Prospective feasibility analysis of a novel off-line approach for MR-guided radiotherapy. *Strahlenther Onkol.* (2018) 194:425–34. 10.1007/s00066-017-1258-y 29349601

[31] van Zijtveld M Dirkx M Breuers M Kuipers R Heijmen B. Evaluation of the ‘dose of the day’ for IMRT prostate cancer patients derived from portal dose measurements and cone-beam CT. *Radiother Oncol.* (2010) 96:172–7. 10.1016/j.radonc.2010.05.015 20580111

[32] Owen R Kron T Foroudi F Milner A Cox J Duchesne G. Interfraction prostate rotation determined from in-room computerized tomography images. *Med Dosim.* (2011) 36:188–94. 10.1016/j.meddos.2010.03.002 21540013

[33] Maeda Y Sato Y Minami H Yasukawa Y Yamamoto K Tamamura H Positioning accuracy and daily dose assessment for prostate cancer treatment using in-room CT image guidance at a proton therapy facility. *Med Phys.* (2018) 45:1832–43. 10.1002/mp.12858 29532489

[34] Wang Y Efstathiou JA Sharp GC Lu HM Ciernik IF Trofimov AV. Evaluation of the dosimetric impact of interfractional anatomical variations on prostate proton therapy using daily in-room CT images. *Med Phys.* (2011) 38:4623–33. 10.1118/1.3604152 21928635 PMC3161503

[35] Ariyaratne H Chesham H Pettingell J Alonzi R. Image-guided radiotherapy for prostate cancer with cone beam CT: dosimetric effects of imaging frequency and PTV margin. *Radiother Oncol.* (2016) 121:103–8. 10.1016/j.radonc.2016.07.018 27576431

[36] Li W Vassil A Godley A Mossolly LM Shang Q Xia P. Using daily diagnostic quality images to validate planning margins for prostate interfractional variations. *J Appl Clin Med Phys.* (2016) 17:61–74. 10.1120/jacmp.v17i3.5923 27167262 PMC5690910

[37] Shelley LEA Scaife JE Romanchikova M Harrison K Forman JR Bates AM Delivered dose can be a better predictor of rectal toxicity than planned dose in prostate radiotherapy. *Radiother Oncol.* (2017) 123:466–71. 10.1016/j.radonc.2017.04.008 28460825 PMC5486775

[38] Tondel H Lund JA Lydersen S Wanderas AD Aksnessaether B Jensen CA Radiotherapy for prostate cancer–does daily image guidance with tighter margins improve patient reported outcomes compared to weekly orthogonal verified irradiation? Results from a randomized controlled trial. *Radiother Oncol.* (2018) 126:229–35. 10.1016/j.radonc.2017.10.029 29398152

[39] Tyagi N Fontenla S Zelefsky M Chong-Ton M Ostergren K Shah N Clinical workflow for MR-only simulation and planning in prostate. *Radiat Oncol.* (2017) 12:119. 10.1186/s13014-017-0854-4 28716090 PMC5513123

[40] de Muinck Keizer DM Pathmanathan AU Andreychenko A Kerkmeijer LGW van der Voort van Zyp JRN Tree AC Fiducial marker based intra-fraction motion assessment on cine-MR for MR-Linac treatment of prostate cancer. *Phys Med Biol.* (2019) 64:07NT02. 10.1088/1361-6560/ab09a6 30794995

[41] Kurz C Suss P Arnsmeyer C Haehnle J Teichert K Landry G Dose-guided patient positioning in proton radiotherapy using multicriteria-optimization. *Z Med Phys.* (2018) 29:216–28. 10.1016/j.zemedi.2018.10.003 30409729

[42] Brand DH Tree AC Ostler P van der Voet H Loblaw A Chu W Intensity-modulated fractionated radiotherapy versus stereotactic body radiotherapy for prostate cancer (PACE-B): acute toxicity findings from an international, randomised, open-label, phase 3, non-inferiority trial. *Lancet Oncol.* (2019) 20:1531–43. 10.1016/S1470-2045(19)30569-8 31540791 PMC6838670

[43] South CP Khoo VS Naismith O Norman A Dearnaley DP. A comparison of treatment planning techniques used in two randomised UK external beam radiotherapy trials for localised prostate cancer. *Clin Oncol (R Coll Radiol).* (2008) 20:15–21. 10.1016/j.clon.2007.10.012 18054471

