# Supplementary figures and images for: Dosimetric Impact of the Positional Imaging Frequency for Hypofractionated Prostate Radiotherapy – A Voxel-by-Voxel Analysis
Source: Front Oncol. 2020 Sep 29;10:564068. doi: 10.3389/fonc.2020.564068 (PMC7550661; doi:10.3389/fonc.2020.564068)

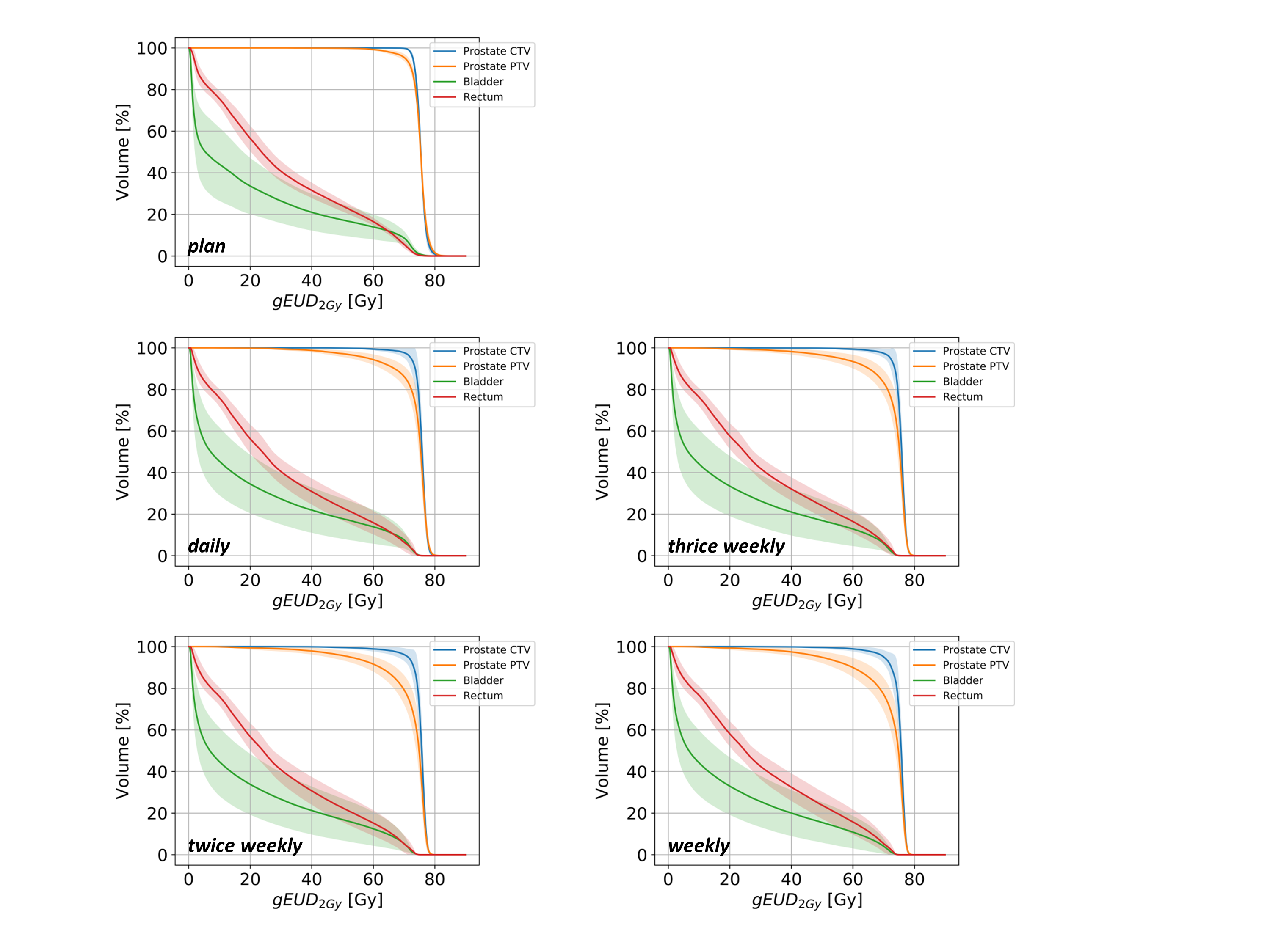

Supplement: Supplementary Figure 1 — Summed dose-volume histograms of the gEUD2Gy for the prostate CTV (blue lines), PTV (orange lines), bladder (green lines) and rectum (red lines) for treatment plans and dose accumulations in dependency of the image-guided repositioning frequency. Lighter-colored bands represent the 95% confidence intervals for each curve. [file Image_1.TIF]
